# Prognostic Value of Tissue Inhibitor of Metalloproteinase-2 Expression in Patients with Non–Small Cell Lung Cancer: A Systematic Review and Meta-Analysis

**DOI:** 10.1371/journal.pone.0124230

**Published:** 2015-04-23

**Authors:** Lin Zhu, Hong Yu, Shi-Yuan Liu, Xiang-Sheng Xiao, Wei-Hua Dong, Yi-Nan Chen, Wei Xu, Tong Zhu

**Affiliations:** 1 Department of Medical imaging, Shanghai Changzheng Hospital affiliated to The Second Military Medical University, Shanghai, China; 2 Department of Periodicals, The Second Military Medical University Library, Shanghai, China; 3 Department of Business Marketing, Shanghai United Imaging Healthcare Co., Ltd, Shanghai, China; Catalan Institute of Oncology, SPAIN

## Abstract

**Background and Objectives:**

Tissue inhibitor of metalloproteinase-2 (TIMP-2) is a small secretory glycoprotein with anti–matrix metalloproteinase activity. Data on the value of TIMP-2 as a prognostic factor in non–small cell lung cancer (NSCLC) are discordant and remain controversial. A systematic review and meta-analysis was performed to explore this issue.

**Methods:**

We identified the relevant literature by searching the PubMed, EMBASE, Web of Science, China National Knowledge Infrastructure, SinoMed, and Wanfang Data databases (search terms: “non-small cell lung cancer” or “NSCLC” or “Lung Carcinoma, Non-Small-Cell”, “Tissue Inhibitor of Metalloproteinase-2” or “TIMP-2”, and “prognosis” or “prognostic” or “survive”) for updates prior to March 1, 2014. The pooled hazard ratio (HR) of overall survival with a 95% confidence interval (95% CI) was used to evaluate the strength of the association between positive TIMP-2 expression and survival in patients with NSCLC.

**Results:**

We included 12 studies in our systematic review; five studies involving 399 patients with NSCLC were meta-analyzed. The pooled HR of all included patients was 0.57 (95% CI: 0.43–0.77), and the HRs of subgroup analysis according to stage (I–IV), testing method (immunohistochemistry) and high TIMP-2 expression percentage (<50%) were 0.63 (95% CI: 0.43–0.92), 0.55 (95% CI: 0.41–0.74), and 0.50 (95% CI: 0.28–0.88), respectively. These data suggested that high TIMP-2 expression is associated with favorable prognosis in NSCLC. The meta-analysis did not reveal heterogeneity or publication bias.

**Conclusions:**

TIMP-2 expression indicates favorable prognosis in patients with NSCLC; as a protective factor, it could help predict outcome and may guide clinical therapy in the future.

## Introduction

The mortality rate of lung cancer (LC) is one of the highest worldwide. Based on GLOBOCAN 2008 estimates, there were an estimated 12.7 million cancer cases and 7.6 million cancer deaths in 2008. Of these, LC was the leading cancer in males, comprising 17% of total new cancer cases and 23% of total cancer deaths [1]. Lozano et al. [2] also reported that 8 million people died from cancer in 2010, the cause of death of 1.5 million of which was tracheal, bronchial, and lung cancer. For treatment purposes, LC is always divided into two major histological subtypes: small cell lung cancer (SCLC) and non–small cell lung cancer (NSCLC) [3]. NSCLC accounts for approximately 85% of LCs, but the 5-year survival rate is only 15%, even though treatments such as surgical management have developed rapidly in recent years [4]. Therefore, diagnosis and treatment at the early stage is important for patients with NSCLC. Understanding the mechanism of invasion and metastasis and exploring means of preventing NSCLC invasion and metastasis at the molecular level is the key to future therapy.

Early studies of NSCLC focused on identifying somatic mutations in genes involved in LC development and led to the discovery of crucial activated oncogenes and abnormal signaling pathways [5]. The development of NSCLC is also considered a multi-step process [6]. In recent years, researchers have realized that NSCLC is not merely caused by gene mutations, but also biological factors in the microenvironment. Currently, much research is focused on this hotspot, and it has been confirmed that many biological factors are associated with NSCLC development and prognosis, such as matrix metalloproteinase (MMP)-2[7], MMP-9 [8], transforming growth factor (TGF) [9], and cyclophilin A (CypA) [10].

MMPs are enzymes that degrade almost all of the protein and collagen in the extracellular matrix (ECM), destroying the histological barrier against tumors and playing a key role in tumor invasion and metastasis [11]. Tissue inhibitor of metalloproteinase (TIMP), an MMP inhibitor, is a promising biomarker for reducing cancer occurrence and improving prognosis. It is a glycoprotein with an apparent molecular size of 28.5 kDa that forms a complex of 1:1 stoichiometry with activated interstitial collagenase [12]. TIMP-2 is a TIMP family member, and is a multifunctional protein that is secreted into the ECM. Increased TIMP-2 levels are considered a favorable prognostic indicator in NSCLC, because they are correlated with the inhibition of endothelial cell proliferation and lung cancer cell angiogenesis in vivo [13]. Therefore, TIMP-2 could have significant prognostic value for patients with NSCLC. At the same time, Zhu et al [14]. and Michael et al [15]. reported that it holds no value as a marker, and results have been contradictory. The difference in findings may be due to individual study limitations such as small sample size and low statistical power. Therefore, we aimed to perform a more precise evaluation of the relationship between TIMP-2 expression and survival in patients with NSCLC through meta-analysis.

## Materials and Methods

### Search strategy and study selection

**Fig 1:**
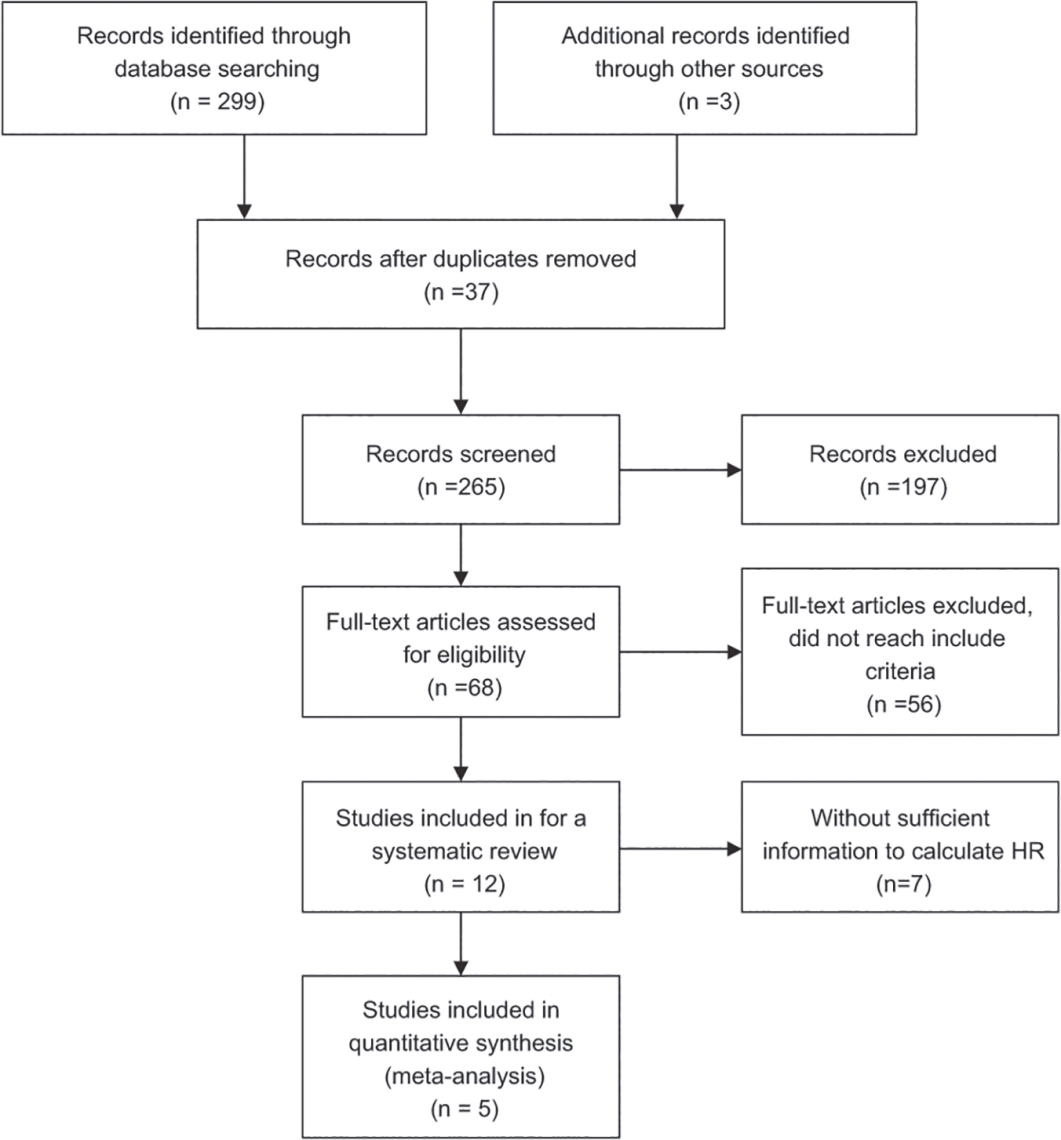
Flowchart of article selection. Flowchart of article selection for systematic review and meta-analysis. HR: hazard ratio.

We searched for relevant studies on TIMP-2 expression and survival in NSCLC patients in the PubMed, EMBASE, Web of Science, China National Knowledge Infrastructure (CNKI), SinoMed, and Wanfang Data databases up until they were updated on March 1, 2014. We also reviewed the reference lists of relevant articles. The search terms were (“non-small cell lung cancer” or “NSCLC” or “Lung Carcinoma, Non-Small-Cell”), (“Tissue Inhibitor of Metalloproteinase-2” or “TIMP-2”), and (“prognosis” or “prognostic” or “survive”). We did not impose language limits on the literature search. To ensure a high-quality systematic review and meta-analysis, we used the following criteria: (1) patients with cytologically or histologically confirmed NSCLC, (2) measured TIMP-2 protein expression, (3) assessed the association between TIMP-2 and survival in NSCLC, (4) a control group to compare survival times between high and low TIMP-2 expression, (5) follow-up was >3 years, (6) for full papers, the hazard ratio (HR) and 95% confidence interval (95% CI) could be obtained from the article or calculated from information therein, (7) in duplicate articles published by the same author, we selected the most complete and newest article. Fig 1 depicts the paper selection process.

### Data extraction

Two authors assessed and selected potential articles independently; disagreements were resolved by debate and discussions with another expert. The data extracted included name of the first author, publication year, number of patients, median or mean patient age, disease stage, TIMP-2 test method, percentage of positive TIMP-2 expression, and estimated HR. Items not included directly in an article were calculated using relevant information provided therein. If the article contained insufficient data, we contacted the authors by email to obtain as much useful information as possible. Items that still could not be obtained were described as “not reported (NR)”.

### Statistical analysis

We used the HR of overall survival and 95% CI to combine the data; if the original article contained the HR and 95% CI, then we used them directly. If not, we calculated the HR and 95% CI using available information in the papers or obtained the information by contacting the authors; Kaplan–Meier survival curves could also be used to estimate a relatively accurate HR [16, 17]. If an article described both univariate and multivariate analysis, such as the report of Lv et al. [18], we chose the latter because survival in NSCLC is affected by a combination of factors. Then, we calculated the logHR and standard error of logHR (SElogHR) of every article and combined them to obtain the HR and 95% CI of all included papers. Heterogeneity was assessed using the I² statistic and chi-square test. If study heterogeneity was considered absent, we used a fixed effects model. By convention, if I² ≥ 50%, heterogeneity was considered present and we used the random effects model instead [19]. Publication bias was assessed using funnel plots; evidence of reliability was derived using sensitivity analysis.

All p-values were 2-sided; p < 0.05 was considered statistically significant. Statistical calculations were performed using Review Manager Version 5.2 provided by the Cochrane Collaboration.

## Results

### Study characteristics

We identified 302 potential articles from the initial search. We excluded 290 articles that did not meet our inclusion criteria. The abstract of one paper, in which the authors stated that they had used tissue microarray and immunohistochemistry (IHC) to detect the expression of 31 molecular markers in patients with NSCLC and had used the Kaplan–Meier method to estimate survival curves, rendered it eligible for inclusion. However, it was a dissertation and we could not find the full text in all of the searched databases for further confirmation, therefore we did not know whether the full details in the article met the inclusion criteria; therefore, we excluded it [20]. We selected 12 articles for the systematic review, as they were full articles and assessed the association between TIMP-2 and survival both in the patients with NSCLC and in the control groups using different methods. At the same time, they followed all included subjects for at least three years. Then, we excluded seven of the 12 eligible studies from the meta-analysis due to insufficient information for calculating the HR. The information provided by two of the excluded articles could only be used to calculate the relative risk (RR) [21, 22]: one found no association between TIMP-2 expression and outcome [21]; the other concluded that TIMP-2 was a harmful factor in NSCLC patients [22]. Four papers reported no significant association between prognosis of NSCLC and TIMP-2 expression [15, 23–25]. By contrast, Yang et al. [26] performed a flow cytometry study and concluded that high TIMP-2 expression was a significant indicator of long survival in patients with NSCLC. Therefore, the remaining five studies with sufficient information for obtaining HR and that involved 399 patients with NSCLC [14, 18, 27–29] were meta-analyzed. They were all published full papers, the sample size of these studies ranged 42–148 patients, and the reported median age was 55–62 years. The percentage of positive TIMP-2 expression ranged from 31% to 67.3%. However, the five included articles all happened to be from Asian countries. Table 1 lists the major characteristics of the eligible studies. We also used the GRADE profiler to evaluate the quality of the included studies, which were all assessed as moderate; Table 2 presents the findings in detail.

**Table 1:** Main characteristics of the eligible studies.

First author	Year	Stage	Median age	No. of patients	Histological type	Ethnicity	Median follow-up (month)	Methods to assess TIMP-2 expression	High expression	HR estimation	HR (95%CI)
Zhu ZH*	2009	IB	56.4	148	NR	Chinese	31	IHC	NR	HR + 95% CI	0.63 (0.37–1.14)
Cao JW*	2010	I-IV(Ⅰ:39 Ⅱ:33 Ⅲ:34 IV:7)	60	113	SCC(65) AdCa(48)	Chinese	33	IHC	76 (67.3%)	Survival curve	0.64 (0.38–1.08)
Lv ZQ*	2006	I-IV(Ⅰ:25 Ⅱ:12 Ⅲ:4 IV:1)	61	42	SCC(15) AC(27)	Chinese	25	IHC	NR	Available data	0.52 (0.27–0.98)
Li HG*	2001	NR	55	42	SCC(15) AC(27)	Chinese	35.5	IHC	13 (31%)	Survival curve	0.39 (0.20–0.77)
Suemitsu R*	2004	I-IV(Ⅰ:31 Ⅱ:3 Ⅲ:18 IV:2)	62	54	SCC(21) AC(33)	Japanese	31.5	ELISA	18 (33.3%)	Survival curve	0.96 (0.33–2.81)
**Zhou JH**	2006	I-III(Ⅰ:11 Ⅱ:21 Ⅲ:11)	57.9	43	SCC(21) AC(22)	Chinese	NA	SP	26 (60.5%)	NA	NA
**Zhang LG**	2004	I-IV(Ⅰ:114 Ⅱ:109 Ⅲ:38 IV:6)	NR	267	SCC(153) AC(114)	Chinese	NA	IHC	63 (19.2%)	NA	NA
**Iniesta P**	2006	I-IV(Ⅰ:67 Ⅱ:8 ⅢA:25 ⅢB:9 IV:2)	63.56	111	SCC(61) AC(46) LCUC(4)	Spanish	60	ELISA	NR	NA	NA
**Michael M**	1999	NR	62.9	46	NR	Canadian	27.5	IHC	> 70%	NA	NA
**Lim BJ**	2009	I-IV(Ⅰ:11 Ⅱ:8 ⅢA:19 ⅢB:3 IV:2)	60.3	43	SCC(31) AC(12)	Korean	65.5	IHC	NR	NA	NA
**Yang DY**	2005	NR	53.5	32	SCC(14) AC(18)	Chinese	30.5	FCM	NR	NA	NA
**Kumaki F**	2001	IA-IIIB(Ⅰ:20 Ⅱ:2 ⅢA:4 ⅢB:1)	66	27	AC(27)	American	60.5	IHC	NR	NA	NA

IHC: Immunohistochemistry; SP: streptavidin–peroxidase; ELISA: enzyme-linked immunosorbent assay; FCM: flow cytometry; TIMP-2: tissue inhibitor of metalloproteinase-2; HR: hazard ratio; CI: confidence interval; NR: not reported; NA: not available; SCC: squamous cell carcinoma; AdCa: adenosquamous carcinoma; AC: Adenocarcinoma; LCUC: Large Cell Carcinoma;

*studies included in the meta-analysis

**Table 2:** GRADE profiler evaluation of the quality of included studies.

Prognostic value of high TIMP-2 expression in patients with NSCLC						
Patient or population: Patients with NSCLC						
Intervention group: High TIMP-2 expression						
Comparison: Low TIMP-2 expression						
Outcomes	Illustrative comparative risks* (95% CI)		Relative effect(95% CI)	No of Participants(studies)	**Quality of the evidence (GRADE)**	Comments
	Assumed risk	Corresponding risk				
**Hazard ratio (HR)**	**Study population**		**HR 0.57**	386	**⊕⊕⊕⊝**	
Dichotomous	**777 per 1000**	**575 per 1000** (476 to 685)	(0.43 to 0.77)	(5 studies)	**Moderate** ^**1**^ ^**,**^ ^**2**^ ^**,**^ ^**3**^ ^**,**^ ^**4**^	
Follow-up: 3 years	**Moderate**					

*The basis for the **assumed risk** (i.e., median control group risk across studies) is described in the footnotes. The **corresponding risk** (and its 95% CI) is based on the assumed risk in the comparison group and the **relative effect** of the intervention (and its 95% CI).

**CI:** Confidence interval; **HR:** Hazard ratio.

GRADE Working Group grades of evidence:

**High quality:** Further research is very unlikely to change our confidence in the estimate of effect.
**Moderate quality:** Further research is likely to have an important impact on our confidence in the estimate of effect and may change the estimate.
**Low quality:** Further research is very likely to have an important impact on our confidence in the estimate of effect and is likely to change the estimate.
**Very low quality:** We are very uncertain about the estimate.

Footnotes:

^1^Negative results without sufficient information.

^2^No unified standard.

^3^Large sample.

^4^Methods used to assess TIMP-2 expression, etc., and multivariate analysis.

### Main meta-analysis results

**Fig 2:**
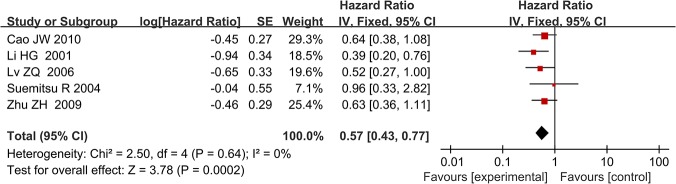
Forest plot of the association between positive TIMP-2 expression and survival in patients with NSCLC. Positive TIMP-2 expression significantly predicted good prognosis in NSCLC (HR = 0.57, P = 0.0002).

**Fig 3:**
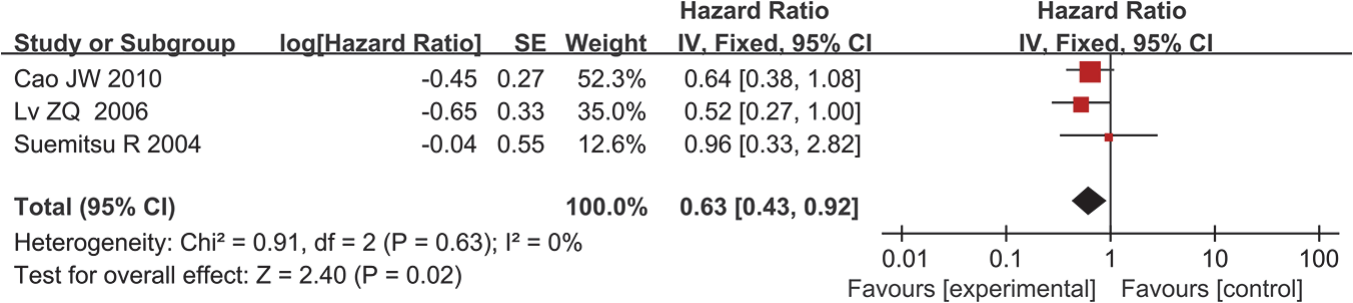
Forest plot of subgroup analysis by disease stage (I–IV). TIMP-2 expression was a statistically significant favorable factor in patients with stage I-IV NSCLC (HR = 0.63, P = 0.02).

**Fig 4:**
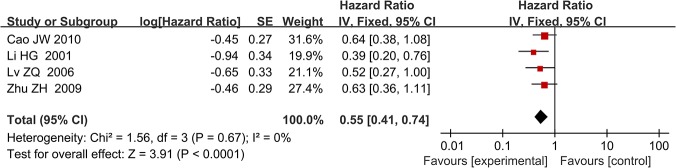
Forest plot of subgroup analysis by test method for TIMP-2 expression (IHC). High TIMP-2 expression remain predicted good prognosis in patients with NSCLC when only IHC was used to assess TIMP-2 expression levels (HR = 0.55, P<0.0001).

**Fig 5:**
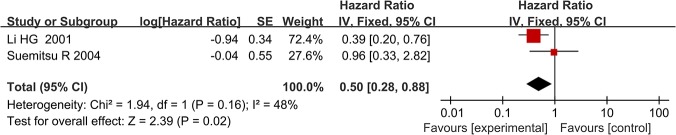
Forest plot of subgroup analysis by percentage of high TIMP-2 expression (<50%). TIMP-2 expression in NSCLC patients was remain a significant predictor of good survival (HR = 0.50, P = 0.02).

Following screening, five studies were included in the final meta-analysis. They had been published between 2001 and 2010. The minimum sample size was 42 and the maximum was 148. The median age of the included patients ranged 55–62 years, and all included patients underwent surgical resection. Most of the included studies reported on stage I–IV disease, but Zhu et al. [14] only included patients with stage IB disease, and Li et al. [28] did not report this information. Specific numbers of included patients with different tumor stages in each article were summarized and listed in Table 1. Four of the five studies were from China, and the remaining study was from Japan. Histological types of most included patients consist of squamous cell carcinoma and adenocarcinoma and histology of patients included in the article from Zhu et al. were not reported. The detail information were summarized in the Table 1. The median follow-up of these studies ranged 25–35.5 years, and the reported percentage of positive TIMP-2 expression was 31–67.3%. Suemitsu et al. [29] sampled blood to perform enzyme-linked immunosorbent assay (ELISA) analysis, and the remaining four studies analyzed tumor and normal tissues using IHC. The HR reported by Lv et al. [18] referred to multivariate analysis, and that of the other four studies referred to univariate analysis. Table 3 summarizes the main meta-analysis results; Figs 2–5 depict the forest plots. The overall combined HR of the included studies was 0.57 (95% CI: 0.43–0.77); we used the fixed effect model because heterogeneity was considered absent (p = 0.64, I² = 0%). This suggests that positive TIMP-2 expression may be a significant predictor of good prognosis in NSCLC.

**Table 3:** Main meta-analysis results.

	No. of studies	No. of included patients	Combined HR (95% CI)	Heterogeneity test (*x^2^*, *p*, *I^2^*)
**Overall**	5	399	0.57 (0.43, 0.77)	2.50, 0.64, 0%
**TIMP-2 in NSCLC tested by IHC**	4	345	0.55 (0.41, 0.74)	1.56, 0.67, 0%
**TIMP-2 in stages I-IV**	3	209	0.63 (0.43, 0.92)	0.91, 0.63, 0%
**TIMP-2 in high expression: <50%**	2	96	0.50 (0.28, 0.88)	1.94, 0.16, 48%

TIMP-2: tissue inhibitor of metalloproteinase-2; HR: hazard ratio; CI: confidence interval; IHC: Immunohistochemistry;

In subgroup analysis, we analyzed the studies according to disease stage (I–IV), testing method for TIMP-2 expression (IHC), and percentage of high TIMP-2 expression (<50%). When we limited the first two subgroup analysis factors, both meta-analyses showed that high TIMP-2 expression improved survival in NSCLC. The combined HRs were 0.63 (95% CI: 0.43–0.92) and 0.55 (95% CI: 0.41–0.74), respectively. When we analyzed the two studies in which the percentage of positive TIMP-2 expression was <50% separately, we found that TIMP-2 still had positive prognostic value in NSCLC; the HR was 0.50 (95% CI: 0.28–0.88). The heterogeneity of every subgroup meta-analysis was non-significant, and we used the fixed effect model for all.

### Publication bias

**Fig 6:**
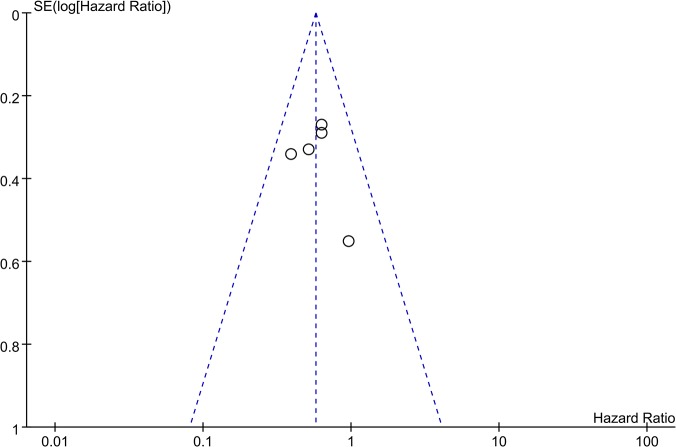
Funnel plot of meta-analysis assessing TIMP-2 expression in patients with NSCLC. Funnel plots were constructed to examine publication bias of all included researches.

Funnel plots were constructed to examine publication bias. The obtained funnel plot presented no proof of obvious publication bias for the included studies (Fig 6).

### Sensitivity analysis

Sensitivity analysis was performed to examine the reliability of our conclusion. We removed one study at a time and the results were not significantly changed (Table 4). This suggests that the conclusion of our meta-analysis is reliable.

**Table 4:** Sensitivity analysis results.

Excluded studies	HR (95% CI)
**Zhu ZH**	0.56 (0.40, 0.78)
**Cao JW**	0.55 (0.39, 0.77)
**Lv ZQ**	0.59 (0.43, 0.81)
**Li HG**	0.63 (0.46, 0.86)
**Suemitsu R**	0.55 (0.41, 0.74)

HR: hazard ratio; CI: confidence interval;

## Discussion

LC, especially NSCLC, is the most common cancer; mortality is high. In recent years, the incidence of LC has increased sharply in both men and women [30]. Therefore, identifying an effective early diagnostic biological marker is vital for patients with NSCLC. At present, the reported biological markers for NSCLC include carcinoembryonic antigen (CEA) [31] and CD133 [32]. A new research hotspot, MMPs destroy the ECM barrier to promote LC angiogenesis and metastasis, and has an unfavorable effect on prognosis [33]. TIMPs are MMP inhibitors that can reduce MMP destruction of the cell matrix and then maintain a complete connection between cells to decrease cancer metastasis and improve prognosis. Some researchers reported that TIMPs induce apoptosis and inhibit invasion by inhibiting signal transducer and activator of transcription-3 (STAT3) by activating the mitogen-activated protein (MAP) kinases such as c-Jun N-terminal kinase (JNK), p38, and extracellular signal–regulated kinase (ERK) in human lung cancer A549 cells [34]. At the same time, Wu et al. [35] reported that when an anti-tumor drug was used on A549 cells, MMP expression was reduced while TIMP-1 and TIMP-2 expression was increased, and the drug suppressed cell invasion and migration by down-regulating MMP-2 and p38 MAPK signaling. Other studies have confirmed that other TIMP family members are associated with cancer prognosis, e.g., TIMP-1 [36, 37] and TIMP-3 [38]. TIMP-2 has garnered much attention in recent years; many researchers have reported its prognostic value in NSCLC. However, the conclusions from individual studies on the relationship between TIMP-2 and NSCLC are inconsistent. Some researchers suggest that it is a protective factor expressed at lower levels in patients with NSCLC, while others have found that expression remains the same or is increased in NSCLC. Therefore, we conducted this meta-analysis to clarify the exact relationship between TIMP-2 and prognosis in NSCLC.

We obtained articles by a comprehensive search strategy and also defined strict inclusion criteria. HR was used as the indicator of time-to-event outcomes for combining individual studies. Compared to the RR or odds ratio, HR measures not only the number of events but also takes into account when they occurred, and is more appropriate for analyzing time-to-event outcomes [16]. The methods for extrapolating the HR are as follows: When the original article did not contain the HR and 95% CI, we calculated them from available data therein or contacted the authors to obtain them. If the data were still insufficient but the original article provided survival curves, we were able to extrapolate the HR using the methods reported by Tierney et al. and Williamson et al. [16, 17]. Although this approach may be less accurate than the methods described earlier, we had two authors read the curve independently to minimize inaccuracy; we found no apparent contradiction when we compared the estimated HRs with the published results in the original articles.

Subgroup analysis is a method for exploring the sources of heterogeneity and for increasing the reliability of an article. Tumor stage is an important factor that affects the prognosis of NSCLC [39]. While the patients in the included studies were of different disease stages, Zhu et al. studied only stage IB NSCLC patients [14], and Li et al. did not report the disease stage of the patients they enrolled [28]. Therefore, we performed subgroup analysis according to stage, and found that TIMP-2 expression was a statistically significant favorable factor in patients with stage I-IV NSCLC. From this, we may assume that high TIMP-2 expression is not only related to outcome in early-stage NSCLC, but also the late stages. The methods used to measure TIMP-2 expression varied between the studies, which may have reduced the reliability of our result. We selected the most commonly applied method, i.e., IHC, for the subgroup analysis and found that the conclusion that high TIMP-2 expression is associated with good prognosis in NSCLC remained the same if only IHC was used to assess TIMP-2 expression levels. The percentage of high TIMP-2 expression of patients in the included studies ranged 31–67.3%. The reasons for this may be the differing states of illness, definitions of positive expression, and race or sex composition of the sampled populations in the included studies. Therefore, we selected studies where the percentage of high TIMP-2 expression was <50% for subgroup analysis to determine the strength of the previous conclusion. This subgroup analysis also suggested that high TIMP-2 expression in NSCLC patients was a significant predictor of good survival. Thus, we believe that TIMP-2 may be a relatively stable indicator of favorable prognosis in NSCLC.

Although we discovered no significant heterogeneity in the included studies, potential heterogeneity we did not discover might still affect the quality of this meta-analysis, and should be taken into account. The possible contributors to heterogeneity are as follows: Although one inclusion criterion was that the time of follow-up must be >3 years, the details of follow-up times in the studies differed; the methods used to assess TIMP-2 expression varied, and even if different studies used the same method, the antibody type and manufacturer would differ; patients selected for each study differed in terms of illness, weight, sex ratio, racial/ethnic composition; each study had its own definition of positive TIMP-2 expression, and there were no unified standards for defining the boundaries of positive expression. When we combined the studies according to the percentage of high TIMP-2 expression being <50%, there was comparatively large heterogeneity (I² = 48%). This may have resulted from the different methods used to assess TIMP-2 expression and the differing patient characteristics.

As an important influencing factor, there has always been publication bias in meta-analysis. This meta-analysis did not detect publication bias, but we realize that we cannot avoid it completely. For example, the included studies were published in different languages, and positive results are likely to be accepted by journals easily; on the contrary, negative results tend to be rejected or are not even submitted. At the same time, a published paper with negative results does not contain detailed data of the negative aspects. We could only include papers that met all of the inclusion criteria but had insufficient data for the systematic review. All this may have contributed to publication bias in our meta-analysis.

To understand our findings better, some limitations should be considered. The disease stage of the included patients was not homogeneous: Zhu et al. [14] studied patients with early-stage disease, and Li et al. [28] did not report this information; Suemitsu et al. [29] performed ELISA analysis on blood samples; another four studies performed IHC analysis of tumor and normal tissues. IHC results are very difficult to reproduce due to several factors, which include the lack of standardized tissue fixation and staining protocols, difficulty in obtaining a consensus standard for microscopic evaluation, and cutoff determination among different laboratories. These factors all could lead to different experimental outcomes. Moreover, four of the included studies used univariate analysis to explore the relationship between TIMP-2 expression and NSCLC outcome [14, 27–29], which is less credible than multivariate analysis such as that by Lv et al. [18]. Multivariate analysis was more important when defining the independent prognostic role of TIMP-2 when assessed together with other potential prognostic factors such as MMP-2 and TIMP-1, as there was likely a certain relationship between the expression of these molecules, and univariate analysis always ignores this. In addition, all patients in the included studies were Asian; the median follow-up duration for each included study differed; the patients included in the five studies all underwent surgical resection and one study [18] reported that the included patients underwent surgical resection but without chemotherapy or radiotherapy; the remaining four studies did not include this information, all of which could have affected survival. Despite our utmost efforts to contact the authors, we were unable to obtain some data on negative results, greatly reducing the number of articles that could have been included in the meta-analysis and details such as disease stage and percentage of high TIMP-2 expression in the included studies were also unknown; moreover, the quality of some of the included studies was not wholly satisfactory. These factors could also have affected the outcome of our evaluation of the prognostic value of TIMP-2, and should not be ignored.

In conclusion, despite the limitations of the present study and heterogeneity across the included studies, our systematic review and meta-analysis suggest that high TIMP-2 expression is a protective factor against the development of NSCLC, and is associated with favorable prognosis in NSCLC. It is a potential new biological marker for the early detection and diagnosis of patients with NSCLC, and can guide clinical therapy in the future or act as a predictor of chemotherapy [40] or a target of inhibitory antibodies [41]. In addition, further investigation involving more high-quality studies and adopting the appropriate multivariate analysis are required to verify and expand on our conclusion.

## Supporting Information

S1 PRISMA Checklist (DOC)
